# Generation of three heterozygous KCNH2 mutation-carrying human induced pluripotent stem cell lines for modeling LQT2 syndrome

**DOI:** 10.1016/j.scr.2021.102402

**Published:** 2021-05-20

**Authors:** Gema Mondéjar-Parreño, James W.S. Jahng, Nadjet Belbachir, Blake C. Wu, Xiaolan Zhang, Marco V. Perez, Nitish Badhwar, Joseph C. Wu

**Affiliations:** a Stanford Cardiovascular Institute, United States; b Depart of Medicine, Division of Cardiovascular Medicine, United States; c Department of Radiology, Stanford University School of Medicine, United States

## Abstract

Congenital long QT syndrome type 2 (LQT2) results from *KCNH2* mutations that cause loss of Kv11.1 channel function which can lead to arrhythmias, syncope, and sudden death. Here, we generated three human-induced pluripotent stem cell (iPSC) lines from peripheral blood mononuclear cells (PBMCs) of two LQT2 patients carrying pathogenic variants (c.1714G > A and c.2960del) and one LQT2 patient carrying a variant of uncertain significance (c.1870A > T) in *KCNH2*. All lines show typical iPSC morphology, high expression of pluripotent markers, normal karyotype, and differentiate into three germ layers *in vitro*. These lines are valuable resources for studying the pathological mechanisms of LQTS caused by caused by *KCNH2* mutations.

## 1. Resource utility

These iPSC lines generated from two individuals carrying pathogenic variants and from an individual carrying a variant of uncertain significance in *KCNH2* gene provide an unlimited source for differentiating iPSC-derived cardiomyocytes (iPSC-CMs) *in vitro*. They are an excellent tool for modelling LQT2 to elucidate underlying pathological mechanisms and for drug screenings (Table 1).

## 2. Resource details

The long QT syndrome (LQTS) is a hereditary arrhythmogenic disorder characterized by a prolongation of the QT interval on electrocardiogram (ECG) that predisposes patients to life-threatening ventricular arrhythmias such as Torsade de Pointes (TdP) and sudden cardiac death (Anderson et al., 2006). The *KCNH2* or *human ether-a-go-go-related* gene 1 (hERG1) encodes the Kv11.1 channel α subunit, which underlies the rapidly activating delayed rectifier K^+^ current (IKr) in the heart during phases 2 and 3 of the cardiac action potential, thus playing an important role in cardiac repolarization (De Zio et al., 2019). LQT2 results from *KCNH2* mutations that cause loss of Kv11.1 channel function (Anderson et al., 2006). Genetic analyses have identified ~200 LQT2-associated *KCNH2* mutations, with autosomal dominant missense (single amino acid substitution) mutations resulting most commonly in protein functional abnormalities (Anderson et al., 2006). Treatment begins with β-blockers unless there are valid contraindications (De Zio et al., 2019).

**Fig. 1.**
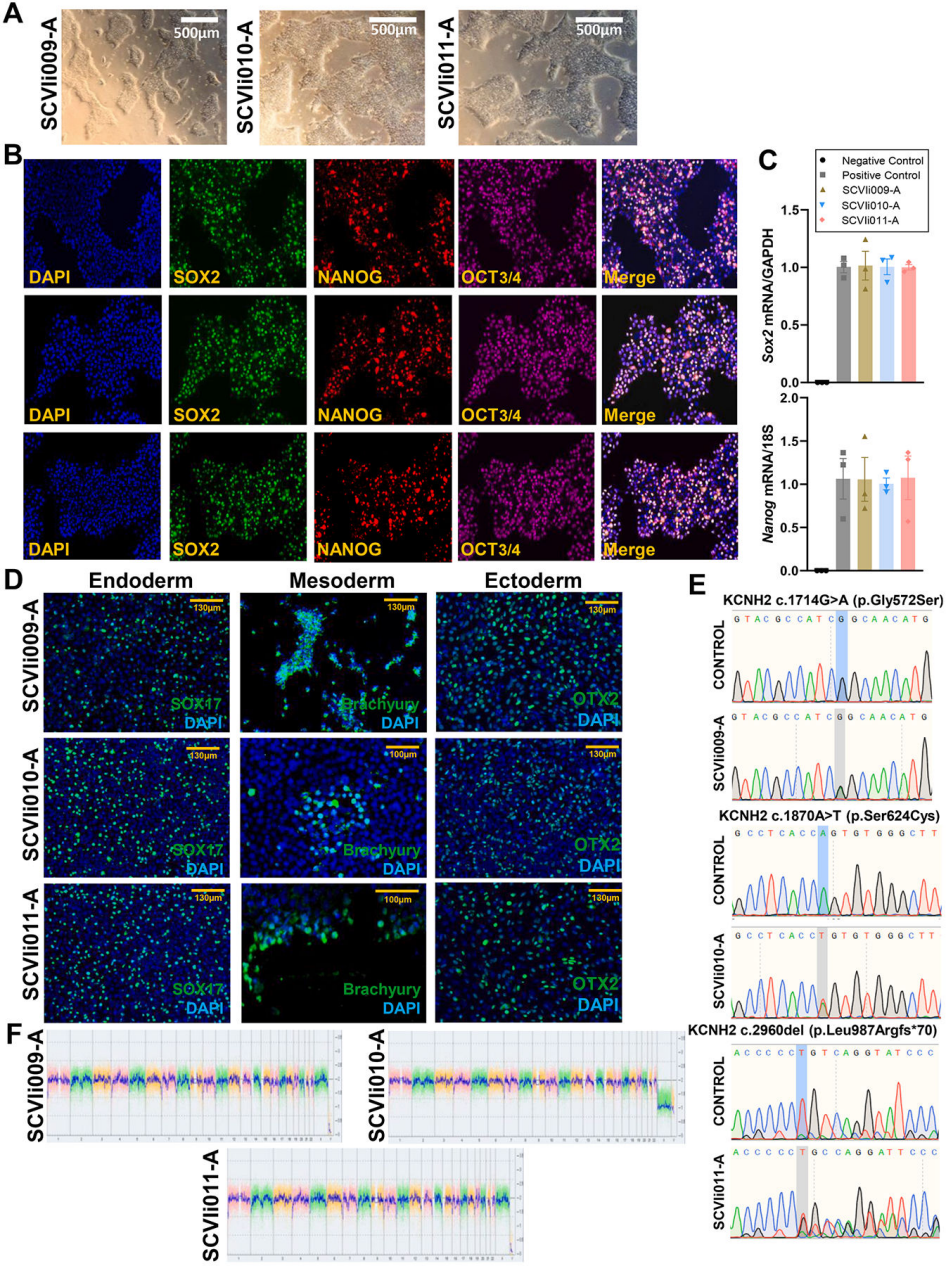


Here, we report three iPSC lines from patients carrying pathogenic variants (c.1714G > A; c.2960del) or a variant of uncertain significance (c.1870A > T) in the *KCNH2* gene, providing a resource for investigating inherited arrhythmia syndromes and testing antiarrhythmic strategies (Garg et al., 2018). The iPSC lines were reprogrammed from peripheral blood mononuclear cells (PBMCs) isolated from an 18-year-old woman with LQT2 (c.1714G > A), a 27-year-old man with LQT2 (c.1870A > T), and a 20-year-old woman with LQT2 (c.2960del) (Table 3). Reprogramming from PBMCs to iPSCs was performed using Sendai virus containing the four Yamanaka factors. The three iPSC clones had normal morphology and karyotype, as evaluated by the *KaryoStat* assay (passages 10 and 13, respectively). High expression of the pluripotency markers OCT3/4, NANOG, and SOX2 was shown by immunostaining. Reverse transcription-quantitative polymerase chain reaction (RT-qPCR) confirmed the comparable expression of Sox2 and Nanog mRNA in these three iPSC lines with that of the widely used positive control iPSC line (Sun et al., 2012) (Fig. 1C). Additionally, the iPSCs were able to differentiate into derivatives of all three germ layers (Fig. 1D). The three heterozygous (c.1714G > A, c.1870A > T, and c.2960del) mutations were confirmed by Sanger sequencing (Fig. 1D and Table 2). All lines were mycoplasma-negative. The origin of these three lines was confirmed by short tandem repeat (STR) analysis which demonstrated that the line profiles completely overlapped with those of the donor somatic cells.

## 3. Materials and methods

### 3.1. Reprogramming

PBMCs were isolated from blood by Percoll^R^ separation, purified using DPBS and plated in a 24-well plate as previously described (Liu et al., 2021). PBMCs (~1–2 × 10^6^) were cultured in 1 mL of complete Stem-Pro^™^-34 medium combined with its specific supplements (in ng/mL): 100 FLT3, 20 IL-6 and 20 EPO, 20 IL-3 and 100 SCF as previously described (Liu et al., 2021). Stem-Pro^™^-34 medium was changed every two days until cell culture stabilization. About 2 × 10^5^ PBMCs resuspended in 300 μl of Stem-Pro^™^-34 medium were infected with a Sendai virus reprogramming cocktail instructions of the CytoTune^®^-iPSC Sendai Reprogramming Kit from Thermo Fisher following manufacturer’s (Liu et al., 2021). After 24 h, cells were replated in one well of a Matrigel-coated 12-well plate. Stem-Pro^™^-34 medium was replaced every two days until day 7, when 1 mL of supplemented StemMACS^™^ iPS-Brew XF medium (Miltenyi Biotec) was added on top of the Stem-Pro^™^-34 medium. On day 8, the medium was replaced with fresh StemMACS^™^ medium. Fresh StemMACS^™^ iPS-Brew XF medium was changed after Sendai virus infection every two days until days 10–15, when colonies appeared and were prepared for picking and expanded as previously described (Liu et al., 2021).

## 4. Cell culture

iPSCs were cultured in sterile conditions using a supplemented StemMACS^™^ iPS-Brew XF medium and changed every other day. At 90% confluency, iPSCs were passaged using 10 μM Y-27632, a potent inhibitor of ROCK1 (Selleck Chemicals), diluted in StemMACS^™^ medium. Y27632 was withdrawn after 24 h. iPSCs were maintained at 37 °C, 85% RH and 5% CO_2_.

## 5. Karyotyping

The whole-genome array for detection of chromosomal abnormalities was performed with KaryoStat^™^ assay from Thermo Fisher in iPSCs at passages 10–13.

## 6. Trilineage differentiation

The STEMdiff^™^ Trilineage Differentiation Kit from STEMCELL Technologies was used to functionally validate the ability of new iPSCs lines to differentiate into the three germ layers following manufacturer’s instructions.

## 7. Immunofluorescent staining

For qualitative analysis of pluripotency and positive three germ layer markers, iPSCs were fixed for 10 min at room temperature using 4% paraformaldehyde. Hereinafter iPSCs were permeabilized 10 min at room temperature using 50 μg/mL digitonin (Sigma Aldrich), and blocked for 30 min at room temperature with 1% of BSA plus 5% of FBS in PBS. iPSCs were incubated overnight at 4 °C with primary antibodies diluted in 1% BSA-PBS (Table 2). At second day, iPSCs were incubated with secondary antibody in 1% BSA-PBS (Table 2) for 30–60 min at room temperature. Nuclei were counterstained using NucBlue^™^ from Invitrogen^™^.

## 8. RT-qPCR

Total RNA was extracted and isolated using the Direct-zol^™^ RNA Miniprep Kit from ZYMO RESEARCH according to manufacturer’s protocol. RT-PCR was performed using iScript^™^cDNA Synthesis Kit from BIORAD using following protocol: 5 min at 25 °C, 20 min at 46 °C, and 1 min at 95 °C. Sox2 and Nanog were amplified using commercial primers (Table 2) and TaqMan^™^ Gene Expression Assay from Applied Biosystems^™^.

## 9. Short tandem repeat analysis (STR)

To verify the origin of these newly generated iPSC lines, gDNA from PBMCs and iPSCs were isolated and purified using DNeasy Blood & Tissue Kit from Qiagen. STR analysis were performed using CLA Identifiler^™^ Plus and Identifiler^™^ Direct PCR Amplification Kits from Thermo Fisher by Stanford PAN Facility.

## 10. Sanger sequencing

PCR primers were designed to amplify a 300- to 500-bp products flanking each *KCNH2* mutations (Table 2). gDNA was isolated from iPSCs using DNeasy Blood & Tissue Kit (Qiagen) following manufacturer’s protocol. *KCNH2* mutation analysis was performed on a purified PCR product obtained from gDNA amplification using NEBNext High-Fidelity (New England Biolabs). PCR product was purified using QIA-quick Purification Kit from Qiagen and sent for Sanger sequencing.

## 11. Mycoplasma detection

Mycoplasma contamination was evaluated using the MycoAlert Detection Kit (Lonza) following manufacturer’s instructions.

## Supplementary Material

supplemental

## Figures and Tables

**Table 1:** Characterization and validation.

Classification	Test	Result	Data
Morphology	Photography Bright field	Normal	Fig. 1A
Phenotype	Qualitative analysis	Positive expression of pluripotency markers: Oct4, Nanog, Sox2	Fig. 1B
	Quantitative analysis	mRNA expression for Sox2 and Nanog	Fig. 1C
Genotype	Whole genome array (KaryoStat^™^ Assay) Resolution 1–2 Mb	*Normal karyotype: 46, XX for SCVIi009-A and SCVIi011-A; 46, XY for SCVIi010-A*	Fig. 1F
Identity	STR analysis	22 loci tested, 100% matching identity	Supplementary Fig. A
Mutation analysis (IF APPLICABLE)	Sequencing	*Heterozygous KCNH2 (c.1714G > A; c.1870A > T; c.2960del) for the three iPSC lines*	Fig. 1E
	Southern Blot OR WGS	*N/A*	*N/A*
Microbiology and virology	Mycoplasma	*Luminescence: negative*	Supplementary Fig. B
Differentiation potential	Qualitative analysis	Positive expression of pluripotency markers: Oct4, Nanog, Sox2	Fig. 1B
	Quantitative analysis	mRNA expression for Sox2 and Nanog	Fig. 1C
List of recommended germ layer markers	Trilineage *in vitro* differentiation by immunofluorescence analysis	*Positive staining of three germ layer markers: ectoderm (OTX2), mesoderm (BRACHYURY), endoderm (SOX17)*	Fig. 1D
Donor screening (OPTIONAL)	HIV 1 + 2 Hepatitis B, Hepatitis C	*Not performed*	Not performed
Genotype additional info (OPTIONAL)	Blood group genotyping	*Not performed*	Not performed
	HLA tissue typing	*Not performed*	Not performed

**Table 2:** Reagents details.

Antibodies and stains used for immunocytochemistry/flow-cytometry			
	Antibody	Dilution	Company Cat # and RRID
Pluripotency Marker	*Mouse IgG2b κ Anti-OCT-3/4*	*1:200*	*Santa Cruz Biotechnology Cat# sc-5279, RRID:AB628051*
Pluripotency Marker	*Rabbit Anti-NANOG*	*1:200*	*Protein tech Cat# 142951-1-AP, RRID:AB1607719*
Pluripotency Marker	*Mouse IgG1 κAnti-SOX2*	*1:200*	*Santa Cruz Biotechnology Cat# sc-365823, RRID: AB_10842165*
Ectoderm Marker	*Goat Anti-OTX2*	*1:200*	*R&D Systems Cat#963273, RRID:AB_2157172*
Endoderm Marker	*Goat Anti-SOX17*	*1:200*	*R&D Systems Cat# 963121, RRID:AB355060*
Mesoderm Marker	*Goat Anti-BRACHYURY*	*1:200*	*R&D Systems Cat#963427, RRID:AB_2200235*
Secondary Antibody	*Alexa Fluor 488 Goat Anti-Mouse IgG1*	*1:1000*	*Thermo Fisher Scientific #A-21121 RRID:AB_2535764*
Secondary Antibody	*Alexa Fluor 647 Goat Anti-Mouse IgG2b*	*1:250*	*Thermo Fisher Scientific #A-21242 RRID:AB2535811*
Secondary Antibody	*Alexa Fluor 555 Goat Anti-Rabbit IgG (H + L)*	*1:500*	*Thermo Fisher Scientific #A-21428 RRID:AB_141784*
Secondary Antibody	*Alexa Fluor 488 Donkey Anti-Goat IgG (H + L)*	*1:1000*	*Thermo Fisher Scientific #A-11055 RRID:AB2534102*
Primers and Oligonucleotides used in this study			
Primers	Target	Forward/Reverse primer (5′-3′)	
Genotyping	*KCNH2 (c.1714G > A)*	*F:CACAGCCAATGAGCATGACG* *R:ATCTGACCTCTGATGCTCGC*	
	*KCNH2 (c.1870A > T)*	*F:GGAGTCTCTAAGTTCCAGGGC* *R:ACTGGCTAGCCTGCATCTG*	
	*KCNH2 (c.2960del)*	*F:GCTGAAAATGTTGGACACTCCTG* *R:GTTTCCCACAGACACGGAGC*	
Pluripotency Markers (qPCR)	*Sox2*	*Hs04234836_s1*	
	*Nanog*	*Hs02387400_g1*	
Housekeeping genes	*GAPDH*	*Hs02786624_g1*	
	*18S*	*Hs03003631_g1*	

**Table 3:** Summary of lines.

iPSC line names	Gender	Age	Ethnicity	Genotype	Clinical disease	Clinician Classification	Clinical presentation
SCVIi009-A	*Female*	*18*	*White*	*c.1714G > A heterozygous*	LQT2	*Pathogenic*	*Syncope in context of poor oral intake and recent upper respiratory infection, and was found to have prolonged QTc with intermittent Torsade de Pointes. No suspicious family cardiac history.*
SCVIi010-A	*Male*	*27*	*White*	*c.1870A > T heterozygous*	LQT2	*Variant of Uncertain Significance*	*Prolonged QT discovered during evaluation for dizziness. No suspicious family cardiac history.*
SCVIi011-A	*Female*	*20*	*Hispanic or Latino*	*c.2960del heterozygous*	LQT2	*Pathogenic*	*Episodes of ventricular fibrillation, longest episode lasting 135 s. Episodes of syncope that started at age 16. Survived cardiac arrest requiring CPR. No suspicious family cardiac history.*

**Table:** Resource Table:

Unique stem cell lines identifiers	1. *SCVIi009-A* 2. *SCVIi010-A* 3. *SCVIi011-A*		
Alternative name(s) of stem	1. *SCVI2177; SCVI-2177* 2. *SCVI2182; SCVI-2182* 3. *SCVI2237; SCVI-2237*		
Institution	*Stanford Cardiovascular Institute*		
Contact information of distributor	*Dr. Joseph C. Wu;* joewu@stanford.edu		
Type of cell lines	*iPSC*		
Origin	*Human*		
Additional origin info required	*SCVIi009-A*	*SCVIi0010-A*	*SCVIi011-A*
	*Age: 18*	*Age: 27*	*Age: 20*
	*Sex: female*	*Sex: male*	*Sex: female*
	*Ethnicity:*	*Ethnicity:*	*Ethnicity:*
	*White*	*White*	*Hispanic/Latino*
Cell Source	*Original cell type induced for reprogramming: blood*		
Clonality	*Clonal*		
Method of reprogramming	*Integration-free Sendai virus expressing human OCT4, SOX2, KLF4, and c-MYC*		
Genetic Modification	*YES*		
Type of Genetic Modification	*Spontaneous mutation*		
Evidence of the reprogramming	*RT-/q-PCR, ICC*		
Associated disease	*Congenital long QT syndrome type 2 (LQT2)*		
Gene/locus	*KCNH2 (7q36.1)*		
	*SCVIi009-A: Heterozygous KCNH2 (c.1714G > A)* *SCVIi010-A: Heterozygous KCNH2 (c.1870A > T)* *SCVIi011-A: Heterozygous KCNH2 (c.2960del)*		
Date archived/stock date			*SCVIi009-A: 6/7/2019* *SCVIi010-A: 5/3/2019* *SCVIi011-A: 12/3/2019*
Cell line repository/bank	https://hpscreg.eu/cell-line/SCVIi009-A https://hpscreg.eu/cell-line/SCVIi010-A https://hpscreg.eu/cell-line/SCVIi011-A		
Ethical approval	*The generation of the lines was approved by the Administrative Panel on Human Subjects in Medical Research, Stanford University under IRB #29904 for working with human subjects.*		

## Data Availability

Data will be made available on request.
