# Supplementary figures and images for: Generation of three heterozygous KCNH2 mutation-carrying human induced pluripotent stem cell lines for modeling LQT2 syndrome
Source: Stem Cell Res. Author manuscript; Available in PMC 2024 Feb 19. (PMC10875632; doi:10.1016/j.scr.2021.102402)

A

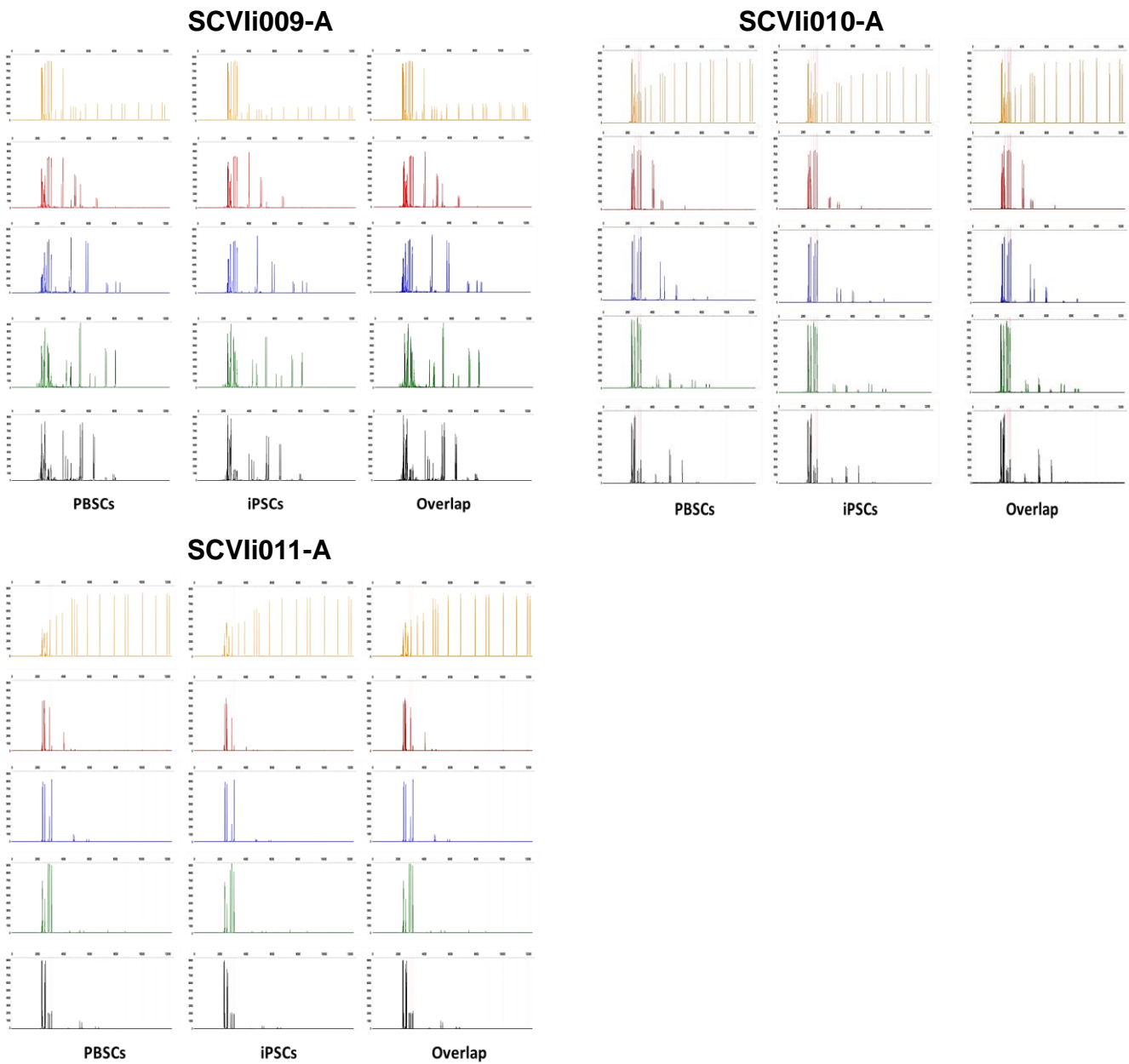

B

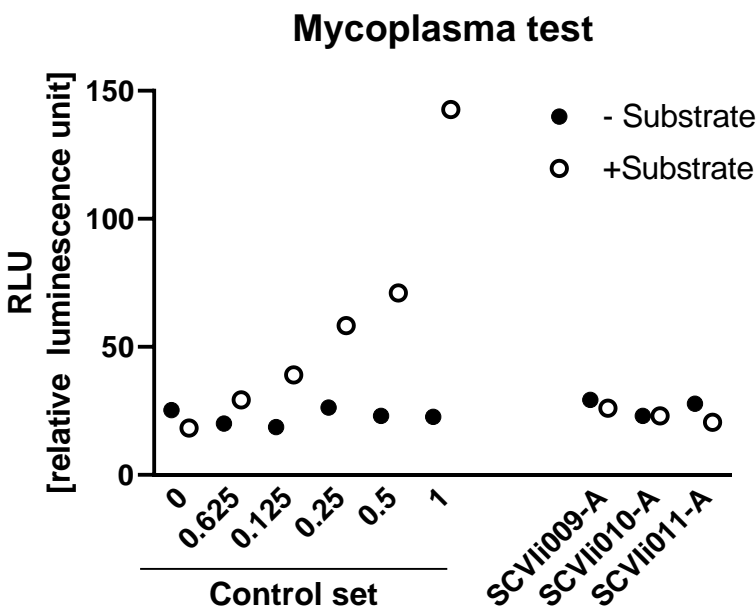

| Values      | Control serial dilution |
|-------------|-------------------------|
| 0.723684211 | 0                       |
| 1.466666667 | 0.625                   |
| 2.089285714 | 0.125                   |
| 2.215189873 | 0.25                    |
| 3.086956522 | 0.5                     |
| 6.294117647 | 1                       |
| 0.79787234  | SCVli009-A              |
| 1.090909091 | SCVli010-A              |
| 0.853658537 | SCVli011-A              |

<1.4 Mycoplasma negative

Supplement: supplemental [file NIHMS1955648-supplement-supplemental.pdf]
